# Supplementary material for: Psychological distress among healthcare providers during COVID-19 in Asia: Systematic review and meta-analysis
Source: PLoS One. 2021 Oct 14;16(10):e0257983. doi: 10.1371/journal.pone.0257983 (PMC8516240; doi:10.1371/journal.pone.0257983)
Supplement: S2 Table — (DOCX) [file pone.0257983.s009.docx]

Table A2: Search terms used from 13^th^ of March to 15^th^ of March 2021

| **Date** | **No** | **CINAHL & MEDLINE** | **Hits CINAHL** | **MEDLINE** |
| --- | --- | --- | --- | --- |
| 15/3/2021 | S1 | TI ( "burned out" OR burnout OR "burning out" OR "burn out" OR anxiety OR "emotional stress” OR "emotional exhaustion" OR "secondary trauma*" OR "vicarious trauma*"OR "compassion fatigue" OR "Psychological Distress" OR Fear OR Resilience OR Adaptation OR Coping OR adjustment ) OR AB ( "burned out" OR burnout OR "burning out" OR "burn out" OR anxiety OR "emotional stress” OR "emotional exhaustion" OR "secondary trauma*" OR "vicarious trauma*"OR "compassion fatigue" OR "Psychological Distress" OR Fear OR Resilience OR Adaptation OR Coping OR adjustment ) | 49,644 | 131,178 |
|  | S2 | (MM "Burnout, Professional") OR (MM "Mental Fatigue") OR (MM "Anxiety") OR (MM "Fear") OR (MM "Hopelessness") OR (MM "Psychological Distress") OR (MM "Frustration") OR (MM "Depression") OR ( (MM "Stress, Occupational") OR (MM "Stress, Psychological") OR (MH "Adaptation, Psychological+") | 22,073 | 26,205 |
|  | S3 | S1 OR S2 | 60,673 | 143,432 |
|  | S4 | TI ( "SARS" OR "COV" OR "coronavirus" OR "MERS" OR "Orthocoronavirinae" OR "Severe Acute Respiratory Syndrome” OR "Middle East Respiratory Syndrome" OR "CoV-19" OR "SARS-CoV" OR "SARS-CoV-2" OR "2019 nCoV" OR "2019nCoV" OR "2019 novel coronavirus" OR "COVID 19" OR "new coronavirus" OR "novel coronavirus" OR "SARS CoV-2" OR "Wuhan coronavirus" OR "COVID 19" OR "2019- nCoV" ) OR AB ( "SARS" OR "COV" OR "coronavirus" OR "MERS" OR "Orthocoronavirinae" OR "Severe Acute Respiratory Syndrome” OR "Middle East Respiratory Syndrome" OR "CoV-19" OR "SARS-CoV" OR "SARS-CoV-2" OR "2019 nCoV" OR "2019nCoV" OR "2019 novel coronavirus" OR "COVID 19" OR "new coronavirus" OR "novel coronavirus" OR "SARS CoV-2" OR "Wuhan coronavirus" OR "COVID 19" OR "2019- nCoV" ) | 18,379 | 73,690 |
|  | S5 | (MM "COVID-19") | 10,657 | 23,318 |
|  | S6 | S4 OR S5 | 25,045 | 85,671 |
|  | S7 | TI ( "professionals" OR "worker*" OR "doctor*" OR "nurse*" OR "occupation*" OR "employee*" OR "healthcare provider*" OR "healthcare worker*" OR "healthcare employee*" OR "personnel" OR "emergency worker" OR "paramedic*" OR "Health care providers" OR "Health care personnel "OR "Health care workers" OR "Health care staff" OR "Health care professionals" OR "Health care staff" OR Doctors OR Nurses OR Physicians OR "Medical personnel" OR Medics OR surgeon OR "hospital personnel" ) OR AB ( "professionals" OR "worker*" OR "doctor*" OR "nurse*" OR "occupation*" OR "employee*" OR "healthcare provider*" OR "healthcare worker*" OR "healthcare employee*" OR "personnel" OR "emergency worker" OR "paramedic*" OR "Health care providers" OR "Health care personnel "OR "Health care workers" OR "Health care staff" OR "Health care professionals" OR "Health care staff" OR Doctors OR Nurses OR Physicians OR "Medical personnel" OR Medics OR surgeon OR "hospital personnel" ) | 841,878 | 1,448,307 |
|  | S8 | (MH "Health Personnel+") | 596,853 | 532,366 |
|  | S9 | S7 OR S8 | 1,208,660 | 1,731,707 |
|  | S10 | TI ( Kazakhstan OR Kyrgyzstan OR Tajikistan OR Turkmenistan OR Uzbekistan OR Borneo OR Brunei OR Cambodia OR "East Timor" OR Indonesia OR Laos OR Malaysia OR Myanmar OR Philippines OR Singapore OR Thailand OR Timor OR Vietnam OR Bangladesh OR Bhutan OR India OR Afghanistan OR Bahrain OR Iran OR Iraq OR Israel OR Jordan OR Kuwait OR Lebanon OR Oman OR Qatar OR "Saudi Arabia" OR Syria OR Turkey OR "United Arab Emirates" OR Yemen OR Nepal OR Pakistan OR "Sri Lanka" OR China OR Tibet OR "Hong Kong" OR Japan OR Macao OR Mongolia OR "North Korea" OR "South Korea" OR Taiwan OR Asia ) OR AB ( Kazakhstan OR Kyrgyzstan OR Tajikistan OR Turkmenistan OR Uzbekistan OR Borneo OR Brunei OR Cambodia OR "East Timor" OR Indonesia OR Laos OR Malaysia OR Myanmar OR Philippines OR Singapore OR Thailand OR Timor OR Vietnam OR Bangladesh OR Bhutan OR India OR Afghanistan OR Bahrain OR Iran OR Iraq OR Israel OR Jordan OR Kuwait OR Lebanon OR Oman OR Qatar OR "Saudi Arabia" OR Syria OR Turkey OR "United Arab Emirates" OR Yemen OR Nepal OR Pakistan OR "Sri Lanka" OR China OR Tibet OR "Hong Kong" OR Japan OR Macao OR Mongolia OR "North Korea" OR "South Korea" OR Taiwan OR Asia ) | 206,781 | 807,994 |
|  | S11 | (MH "Asia+") | 296,262 | 872,887 |
|  | S12 | S10 OR S11 | 357,265 | 1,222,582 |
|  | S13 | S3 AND S6 AND S9 AND S12  Published Date: 20190101-20211231 | 155 | 500 |
|  |  | **Pubmed** | **Hits** |  |
| 13/3/2021 |  | (((("burned out"[Title/Abstract] OR burnout[Title/Abstract] OR "burning out"[Title/Abstract] OR "burn out" [Title/Abstract] OR anxiety[Title/Abstract] OR "emotional stress"[Title/Abstract] OR "emotional exhaustion" OR "secondary trauma*"[Title/Abstract] OR "vicarious trauma*"OR "compassion fatigue"[Title/Abstract] OR "Psychological Distress"[Title/Abstract] OR Fear[Title/Abstract] OR Resilience[Title/Abstract] OR Adaptation[Title/Abstract] OR Coping[Title/Abstract] OR adjustment[Title/Abstract]) OR ((((((((burnout, professional[MeSH Terms]) OR (Fatigue[MeSH Terms])) OR (Fear[MeSH Terms])) OR (Anxiety[MeSH Terms])) OR (Depression[MeSH Terms])) OR (stress, psychological[MeSH Terms])) OR (Resilience, Psychological[MeSH Terms])) OR (Adaptation, Psychological[MeSH Terms]))) AND (("SARS" [Title/Abstract] OR "COV"[Title/Abstract] OR "coronavirus" [Title/Abstract] OR "MERS"[Title/Abstract] OR "Orthocoronavirinae" [Title/Abstract] OR "Severe Acute Respiratory Syndrome"[Title/Abstract] OR "Middle East Respiratory Syndrome"[Title/Abstract] OR "CoV-19"[Title/Abstract] OR "SARS-CoV"[Title/Abstract] OR "SARS-CoV-2"[Title/Abstract] OR "2019 nCoV"[Title/Abstract] OR "2019nCoV"[Title/Abstract] OR "2019 novel coronavirus"[Title/Abstract] OR "COVID 19"[Title/Abstract] OR "new coronavirus"[Title/Abstract] OR "novel coronavirus"[Title/Abstract] OR "SARS CoV-2""Wuhan coronavirus"[Title/Abstract] OR "COVID 19"[Title/Abstract] OR "2019-nCoV"[Title/Abstract]) OR ((COVID-19[MeSH Terms]) OR (SARS-CoV-2[MeSH Terms])))) AND (("professionals"[Title/Abstract] OR "worker*"[Title/Abstract] OR "doctor*"[Title/Abstract] OR "nurse*"[Title/Abstract] OR "occupation*"[Title/Abstract] OR "employee*"[Title/Abstract] OR "healthcare provider*"[Title/Abstract] OR "healthcare worker*"[Title/Abstract] OR "healthcare employee*"[Title/Abstract] OR "personnel"[Title/Abstract] OR "emergency worker"[Title/Abstract] OR "paramedic*"[Title/Abstract] OR "Health care providers"[Title/Abstract] OR "Health care personnel "OR "Health care workers"[Title/Abstract] OR "Health care staff"[Title/Abstract] OR "Health care professionals"[Title/Abstract] OR "Health care staff"[Title/Abstract] OR Doctors[Title/Abstract] OR Nurses [Title/Abstract] OR Physicians[Title/Abstract] OR "Medical personnel"[Title/Abstract] OR Medics[Title/Abstract] OR surgeon[Title/Abstract] OR "hospital personnel"[Title/Abstract]) OR (Health Personnel[MeSH Terms]))) AND ((Kazakhstan[Title/Abstract] OR Kyrgyzstan[Title/Abstract] OR Tajikistan[Title/Abstract] OR Turkmenistan[Title/Abstract] OR Uzbekistan[Title/Abstract] OR Borneo[Title/Abstract] OR Brunei[Title/Abstract] OR Cambodia[Title/Abstract] OR "East Timor"[Title/Abstract] OR Indonesia[Title/Abstract] OR Laos[Title/Abstract] OR Malaysia[Title/Abstract] OR Myanmar[Title/Abstract] OR Philippines[Title/Abstract] OR Singapore[Title/Abstract] OR Thailand[Title/Abstract] OR Timor[Title/Abstract] OR Vietnam[Title/Abstract] OR Bangladesh[Title/Abstract] OR Bhutan[Title/Abstract] OR India[Title/Abstract] OR Afghanistan[Title/Abstract] OR Bahrain[Title/Abstract] OR Iran[Title/Abstract] OR Iraq[Title/Abstract] OR Israel[Title/Abstract] OR Jordan[Title/Abstract] OR Kuwait[Title/Abstract] OR Lebanon[Title/Abstract] OR Oman[Title/Abstract] OR Qatar[Title/Abstract] OR "Saudi Arabia"[Title/Abstract] OR Syria[Title/Abstract] OR Turkey[Title/Abstract] OR "United Arab Emirates"[Title/Abstract] OR Yemen[Title/Abstract] OR Nepal[Title/Abstract] OR Pakistan[Title/Abstract] OR "Sri Lanka"[Title/Abstract] OR China[Title/Abstract] OR Tibet[Title/Abstract] OR "Hong Kong"[Title/Abstract] OR Japan[Title/Abstract] OR Macao[Title/Abstract] OR Mongolia[Title/Abstract] OR "North Korea"[Title/Abstract] OR "South Korea"[Title/Abstract] OR Taiwan[Title/Abstract] OR Asia[Title/Abstract]) OR (asia[MeSH Terms])) | 746 |  |
|  |  | S**copus** | **Hits** |  |
| 13/3/2021 |  | TITLE-ABS-KEY ( ( "professionals"  OR  "worker*"  OR  "doctor*"  OR  "nurse*"  OR  "occupation*"  OR  "employee*"  OR  "healthcare provider*"  OR  "healthcare worker*"  OR  "healthcare employee*"  OR  "personnel"  OR  "emergency worker"  OR  "paramedic*"  OR  "Health care providers"  OR  "Health care personnel "  OR  "Health care workers"  OR  "Health care staff"  OR  "Health care professionals"  OR  "Health care staff"  OR  doctors  OR  nurses  OR  physicians  OR  "Medical personnel"  OR  medics  OR  surgeon  OR  "hospital personnel" ) ) | 3815613 |  |
|  |  | TITLE-ABS-KEY ( ( kazakhstan  OR  kyrgyzstan  OR  tajikistan  OR  turkmenistan  OR  uzbekistan  OR  borneo  OR  brunei  OR  cambodia  OR  "East Timor"  OR  indonesia  OR  laos  OR  malaysia  OR  myanmar  OR  philippines  OR  singapore  OR  thailand  OR  timor  OR  vietnam  OR  bangladesh  OR  bhutan  OR  india  OR  afghanistan  OR  bahrain  OR  iran  OR  iraq  OR  israel  OR  jordan  OR  kuwait  OR  lebanon  OR  oman  OR  qatar  OR  "Saudi Arabia"  OR  syria  OR  turkey  OR  "United Arab Emirates"  OR  yemen  OR  nepal  OR  pakistan  OR  "Sri Lanka"  OR  china  OR  tibet  OR  "Hong Kong"  OR  japan  OR  macao  OR  mongolia  OR  "North Korea"  OR  "South Korea"  OR  taiwan  OR  asia ) ) | 4078202 |  |
|  |  | TITLE-ABS-KEY ( covid  19  OR  sars-cov-2  OR  coronavirus ) | 109.558 |  |
|  |  | TITLE-ABS-KEY ( ( "burned out"  OR  burnout  OR  "burning out"  OR  "burn out"  OR  anxiety  OR  "emotional stress"  OR  "emotional exhaustion"  OR  "secondary trauma*"  OR  "vicarious trauma*"  OR  "compassion fatigue"  OR  "Psychological Distress"  OR  fear  OR  resilience  OR  adaptation  OR  coping  OR  adjustment ) ) | 1825884 |  |
|  |  | ( TITLE-ABS-KEY ( ( "professionals"  OR  "worker*"  OR  "doctor*"  OR  "nurse*"  OR  "occupation*"  OR  "employee*"  OR  "healthcare provider*"  OR  "healthcare worker*"  OR  "healthcare employee*"  OR  "personnel"  OR  "emergency worker"  OR  "paramedic*"  OR  "Health care providers"  OR  "Health care personnel "  OR  "Health care workers"  OR  "Health care staff"  OR  "Health care professionals"  OR  "Health care staff"  OR  doctors  OR  nurses  OR  physicians  OR  "Medical personnel"  OR  medics  OR  surgeon  OR  "hospital personnel" ) ) )  AND  ( TITLE-ABS-KEY ( ( "burned out"  OR  burnout  OR  "burning out"  OR  "burn out"  OR  anxiety  OR  "emotional stress"  OR  "emotional exhaustion"  OR  "secondary trauma*"  OR  "vicarious trauma*"  OR  "compassion fatigue"  OR  "Psychological Distress"  OR  fear  OR  resilience  OR  adaptation  OR  coping  OR  adjustment ) ) )  AND  ( TITLE-ABS-KEY ( ( kazakhstan  OR  kyrgyzstan  OR  tajikistan  OR  turkmenistan  OR  uzbekistan  OR  borneo  OR  brunei  OR  cambodia  OR  "East Timor"  OR  indonesia  OR  laos  OR  malaysia  OR  myanmar  OR  philippines  OR  singapore  OR  thailand  OR  timor  OR  vietnam  OR  bangladesh  OR  bhutan  OR  india  OR  afghanistan  OR  bahrain  OR  iran  OR  iraq  OR  israel  OR  jordan  OR  kuwait  OR  lebanon  OR  oman  OR  qatar  OR  "Saudi Arabia"  OR  syria  OR  turkey  OR  "United Arab Emirates"  OR  yemen  OR  nepal  OR  pakistan  OR  "Sri Lanka"  OR  china  OR  tibet  OR  "Hong Kong"  OR  japan  OR  macao  OR  mongolia  OR  "North Korea"  OR  "South Korea"  OR  taiwan  OR  asia ) ) )  AND  ( TITLE-ABS-KEY ( covid  19  OR  sars-cov-2  OR  coronavirus ) ) | 1045 |  |
