# Supplementary material for: Psychological distress among healthcare providers during COVID-19 in Asia: Systematic review and meta-analysis
Source: PLoS One. 2021 Oct 14;16(10):e0257983. doi: 10.1371/journal.pone.0257983 (PMC8516240; doi:10.1371/journal.pone.0257983)
Supplement: S4 Table — (DOCX) [file pone.0257983.s011.docx]

Table A4: Characteristics of 148 studies

| No. | Author | Year | Country | Strobe | Method of screening | | | | | | HCPs | Characteristics of HCPs | | Psychological distresses (Mild and above grade) | | | | | |
| --- | --- | --- | --- | --- | --- | --- | --- | --- | --- | --- | --- | --- | --- | --- | --- | --- | --- | --- | --- |
|  |  |  |  | score | Depression | Anxiety | Stress | Fear | Burnout | Neg Coping | No. of participants | Age | Male, n (%); female, n (%) | Depression, n | Anxiety, n | Stress, n | Fear, n | Burnout,n | Neg Coping, n |
| 1 | Cravero et al | 2020 | Asian Pacific* | 16 | NA | NA | NA | NA | MBI | NA | 301 | NA | NA | NA | NA | NA | NA | 182 | NA |
| 2 | Chew et al A | 2020 | Asian Pacific** | 14 | DASS-21 | DASS-21 | NA | NA | NA | NA | 1146 | 31.7 (7.8) | 400 (34.9); 746 (65.1) | 51 | 60 | NA | NA | NA | NA |
| 3 | Barua et al | 2020 | Bangladesh | 17 | PHQ‐2 | GAD‐2 | NA | FCV-19S | NA | NA | 370 | 30.5 (4.4) | 223 (60.3); 147 (39.7) | 142 | 135 | NA | 370 | NA | NA |
| 4 | Hasan et al M | 2020 | Bangladesh | 14 | HADS | HADS | NA | NA | NA | NA | 412 | NA | 182 (44.2); 230 (55.8) | 200 | 279 | NA | NA | NA | NA |
| 5 | An et al | 2020 | China | 15 | PHQ-9 | NA | NA | NA | NA | NA | 1103 | 32.2 (7.6) | 102(9.2); 1001(90.8) | 481 | NA | NA | NA | NA | NA |
| 6 | Cai et al | 2020 | China | 12 | PHQ-9 | GAD-7 | NA | NA | NA | NA | 709 | NA | 25 (3.5); 684 (96.5) | 374 | 333 | NA | NA | NA | NA |
| 7 | Cao et al | 2020 | China | 15 | HAMD | HAMA | NA | NA | NA | NA | 480 | 35.3 (8.9) | 86 (17.9); 394 (82.1) | 192 | 116 | NA | NA | NA | NA |
| 8 | Chen et al H | 2020 | China | 10 | PHQ‐9 | GAD‐7 | NA | NA | NA | NA | 171 | NA | 55 (32.2); 116 (67.8) | 99 | 95 | NA | NA | NA | NA |
| 9 | Chen et al J | 2021 | China | 14 | PHQ‐9 | GAD‐7 | NA | NA | NA | NA | 902 | NA | 283 (31.4); 619 (68.6) | 439 | 424 | NA | NA | NA | NA |
| 10 | Dong et al | 2020 | China | 14 | NA | NA | NA | NA | NA | NA | 4618 | NA | 755 (16.3); 3863 (83.7) | NA | NA | NA | NA | NA | NA |
| 11 | Fang et al | 2021 | China | 14 | SDS | NA | NA | NA | NA | NA | 511 | 31.2 (6.6) | 88 (17.2); 423 (82.8) | 186 | NA | NA | NA | NA | NA |
| 12 | Gu et al | 2020 | China | 14 | PHQ‐9 | GAD‐7 | PSS-10 | NA | NA | NA | 522 | NA | 117 (22.4); 405 (77.6) | 303 | 395 | 420 | NA | NA | NA |
| 13 | Guo et al | 2021 | China | 12 | DASS-21 | DASS-21 | DASS-21 | NA | NA | NA | 280 | 32.6 (0.5) | NA | 58 | 57 | 27 | NA | NA | NA |
| 14 | He et al | 2021 | China | 13 | PHQ‐9 | GAD‐7 | NA | NA | NA | NA | 403 | NA | 91 (22.6); 312 (77.4) | 196 | 144 | NA | NA | NA | NA |
| 15 | Hu et al D | 2020 | China | 17 | SDS | SAS | NA | FS-HPs | NA | NA | 2014 | 31.0 (6.2) | 260 (12.9); 1754 (87.1) | 878 | 833 | NA | 1837 | NA | NA |
| 16 | Hu et al Z | 2021 | China | 16 | NA | NA | NA | NA | MBI | NA | 2411 | NA | 755 (31.3); 1656 (68.7) | NA | NA | NA | NA | 1681 | NA |
| 17 | Huang et al | 2020 | China | 16 | NA | SAS | NA | NA | NA | CD-RISC | 364 | 32 (27-40) | 150 (41.2); 214 (58.8) | NA | 85 | NA | NA | NA | 59 |
| 18 | Jo et al | 2020 | China | 12 | MINI | MINI | NA | NA | NA | NA | 253 | 39.1 (12.3) | 43 (17.0); 210 (83.0) | 3 | 11 | NA | NA | NA | NA |
| 19 | Juan et al | 2020 | China | 14 | PHQ‐9 | GAD‐7 | NA | NA | NA | NA | 456 | 30.7 (7.5) | 134 (29.4); 322 (70.6) | 135 | 144 | NA | NA | NA | NA |
| 20 | Lai et al | 2020 | China | 16 | PHQ‐9 | GAD‐7 | NA | NA | NA | NA | 1257 | NA | 293 (23.3); 964 (76.7) | 634 | 560 | NA | NA | NA | NA |
| 21 | Li et al A | 2020 | China | 14 | SDS | SAS | NA | NA | NA | NA | 908 | 33.8 (6.9) | 222 (24.4); 686 (75.6) | 299 | 221 | NA | NA | NA | NA |
| 22 | Li et al B | 2020 | China | 14 | NA | DASS-21 | DASS-21 | NA | NA | NA | 225 | NA | 63 (28.0); 162 (72.0) | 105 | 80 | 36 | NA | NA | NA |
| 23 | Li et al C | 2020 | China | 15 | PHQ‐9 | GAD‐7 | NA | NA | NA | NA | 197 | NA | 48 (24.4); 149 (75.6) | 82 | 85 | NA | NA | NA | NA |
| 24 | Li et al D | 2020 | China | 12 | NA | GAD‐7 | NA | NA | NA | NA | 606 | 35.8 (8.1) | 114 (18.8); 492 (81.2) | NA | 275 | NA | NA | NA | NA |
| 25 | Liang et al | 2020 | China | 14 | PHQ‐9 | GAD‐7 | NA | NA | NA | NA | 899 | NA | 168 (18.7); 731 (81.3) | 742 | 552 | NA | NA | NA | NA |
| 26 | Liu et al A | 2020 | China | 14 | NA | SAS | NA | NA | NA | NA | 512 | NA | 79 (15.4); 433 (84.6) | NA | 64 | NA | NA | NA | NA |
| 27 | Liu et al B | 2020 | China | 14 | NA | NA | NA | NA | MBI | NA | 880 | NA | 279 (31.7); 601 (68.3) | NA | NA | NA | NA | 651 | NA |
| 28 | Liu et al C | 2020 | China | 16 | DASS-21 | DASS-21 | DASS-21 | NA | NA | NA | 2031 | NA | 294 (14.5); 1737 (85.5) | 301 | 372 | 203 | NA | NA | NA |
| 29 | Liu et al D | 2020 | China | 16 | NA | NA | NA | NA | NA | NA | 1364 | 30 (27-34) | 292 (21.4); 1072 (78.6) | NA | NA | NA | NA | NA | NA |
| 30 | Lu et al A | 2020 | China | 15 | PHQ‐9 | GAD‐7 | NA | NA | NA | NA | 382 | NA | 146 (38.2); 236 (61.8) | 221 | 214 | NA | NA | NA | NA |
| 31 | Lu et al B | 2020 | China | 16 | HAMD | HAMA | NA | NA | NA | NA | 2299 | NA | 514 (22.4); 1785 (77.6) | 268 | 569 | NA | NA | NA | NA |
| 32 | Mi et al | 2021 | China | 12 | PHQ-4 | PHQ-4 | NA | NA | NA | NA | 1029 | 38.4 (9.2) | 395 (38.4); 634 (61.6) | 137 | 68 | NA | NA | NA | NA |
| 33 | Ni et al | 2020 | China | 13 | PHQ-4 | PHQ-4 | NA | NA | NA | NA | 214 | NA | 67 (31.2); 147 (68.8) | 41 | 47 | NA | NA | NA | NA |
| 34 | Ning et al | 2020 | China | 17 | SDS | SAS | NA | NA | NA | NA | 612 | NA | 166 (27.1); 446 (72.9) | 153 | 100 | NA | NA | NA | NA |
| 35 | Pan et al | 2020 | China | 18 | PHQ‐9 | GAD‐7 | NA | NA | NA | NA | 194 | NA | 36 (18.6); 158 (81.4) | 73 | 63 | NA | NA | NA | NA |
| 36 | Que et al | 2020 | China | 16 | PHQ‐9 | GAD‐7 | NA | NA | NA | NA | 2285 | 31.1 (7.0) | 707 (30.9); 1578 (69.1) | 1014 | 1052 | NA | NA | NA | NA |
| 37 | Ruilin et al | 2020 | China | 14 | NA | HAMA | NA | NA | NA | NA | 176 | NA | 40 (22.7); 136 (77.3) | NA | 136 | NA | NA | NA | NA |
| 38 | Shen et al A | 2020 | China | 14 | NA | SAS | NA | NA | NA | NA | 1637 | NA | 166 (10.1); 1471 (89.9) | NA | 164 | NA | NA | NA | NA |
| 39 | Shen et al B | 2021 | China | 17 | NA | GAD‐7 | NA | NA | NA | NA | 643 | 31.8 (7.8) | 14 (2.2); 629 (97.8) | NA | 215 | NA | NA | NA | NA |
| 40 | Si et al | 2020 | China | 14 | DASS-21 | DASS-21 | DASS-21 | NA | NA | NA | 863 | NA | 253 (29.3); 610 (70.7) | 117 | 120 | 74 | NA | NA | NA |
| 41 | Song et al | 2020 | China | 15 | CES-D | NA | NA | NA | NA | NA | 14825 | 34.0 (8.2) | 5289 (35.7); 9536 (64.3) | 3733 | NA | NA | NA | NA | NA |
| 42 | Sui et al | 2021 | China | 14 | CCMD-3 | CCMD-3 | NA | CCMD-3 | NA | NA | 339 | NA | 32(9.4); 307 (90.6) | 48 | 48 | NA | 53 | NA | NA |
| 43 | Sun et al A | 2021 | China | 16 | DASS-21 | DASS-21 | DASS-21 | NA | NA | NA | 170 | NA | 13 (7.6); 157 (92.4) | 12 | 51 | 29 | NA | NA | NA |
| 44 | Sun et al B | 2020 | China | 16 | PHQ‐9 | GAD‐7 | NA | NA | NA | NA | 536 | 39.2 (9.6) | 166 (31.0); 370 (69.0) | 101 | 52 | NA | NA | NA | NA |
| 45 | Tam et al | 2021 | China | 12 | NA | NA | NA | NA | NA | NA | 1029 | 38.4 (9.2) | 395 (38.4); 634 (61.6) | NA | NA | NA | NA | NA | NA |
| 46 | Teng et al | 2020 | China | 11 | PHQ‐9 | SAS | NA | NA | NA | NA | 398 | NA | 96 (24.1); 302 (75.9) | 194 | 56 | NA | NA | NA | NA |
| 47 | Tu et al | 2020 | China | 13 | PHQ‐9 | GAD‐7 | NA | NA | NA | NA | 100 | 34.4 (5.9) | 0 (0.0);  100 (100.0) | 46 | 40 | NA | NA | NA | NA |
| 48 | Wang et al A | 2020 | China | 14 | HADS | HADS | NA | NA | NA | NA | 1049 | NA | 148 (14.2); 897 (85.8) | 412 | 499 | NA | NA | NA | NA |
| 49 | Wang et al B | 2021 | China | 14 | PHQ‐9 | GAD‐7 | NA | NA | NA | NA | 3460 | NA | 817 (23.6); 2643 (76.4) | 1730 | 1550 | NA | NA | NA | NA |
| 50 | Wang et al C | 2021 | China | 12 | NA | NA | NA | NA | NA | NA | 431 | NA | 94 (21.8); 337 (78.2) | NA | NA | NA | NA | NA | NA |
| 51 | Wang et al D | 2021 | China | 14 | PHQ‐9 | GAD‐7 | NA | NA | NA | NA | 586 | 31.1 (7.5) | 23 (3.9);  563 (96.1) | 192 | 162 | NA | NA | NA | NA |
| 52 | Wang et al E | 2020 | China | 14 | HADS | HADS | NA | NA | NA | NA | 1514 | 33 (29-40) | 517 (34.1); 997 (65.9) | 546 | 354 | NA | NA | NA | NA |
| 53 | Wang et al F | 2020 | China | 16 | NA | NA | NA | NA | NA | NA | 2393 | NA | NA | NA | NA | NA | NA | NA | NA |
| 54 | Wang et al G | 2020 | China | 13 | SDS | SAS | NA | NA | NA | NA | 123 | 33.8 (8.4) | 12 (9.8); 111(90.2) | 31 | 9 | NA | NA | NA | NA |
| 55 | Xia et al | 2021 | China | 10 | PHQ‐9 | Asi-3 | NA | NA | NA | NA | 126 | 35.0 (8.0) | 39 (31.0);  87 (69.0) | 19 | 21 | NA | NA | NA | NA |
| 56 | Xiao et al | 2020 | China | 13 | HADS | HADS | NA | NA | NA | NA | 958 | NA | 314 (32.8); 644 (67.2) | 549 | 518 | NA | NA | NA | NA |
| 57 | Xiaoming et al | 2020 | China | 14 | PHQ‐9 | GAD‐7 | NA | NA | NA | NA | 8817 | 33.3 (8.3) | 6874 (78.0); 1943 (22.0) | 2666 | 1824 | NA | NA | NA | NA |
| 58 | Xing et al A | 2020 | China | 11 | SCL-90 Inventory | SCL-90 Inventory | NA | NA | NA | NA | 548 | NA | 153 (27.9); 395 (72.1) | 163 | 187 | NA | NA | NA | NA |
| 59 | Xing et al B | 2020 | China | 15 | SDS | SAS | NA | NA | NA | NA | 309 | 33.5 (9.5;26-41) | 8 (2.6);  301 (97.4) | 173 | 88 | NA | NA | NA | NA |
| 60 | Xiong et al | 2020 | China | 13 | PHQ‐9 | GAD‐7 | NA | NA | NA | NA | 223 | NA | 6 (2.7);  217 (97.3) | 59 | 91 | NA | NA | NA | NA |
| 61 | Zhan et al A | 2020 | China | 14 | NA | NA | PSS | NA | NA | NA | 1794 | NA | 54 (3.0); 1740 (97.0) | NA | NA | 789 | NA | NA | NA |
| 62 | Zhan et al B | 2020 | China | 14 | PHQ‐9 | GAD‐7 | PSS | NA | NA | NA | 2667 | 30 (26, 35) | 81 (3.0); 2586 (97.0) | 1458 | 1062 | 1298 | NA | NA | NA |
| 63 | Zhang et al A | 2021 | China | 14 | Symptom Checklist 90 | Symptom Checklist 90 | NA | NA | NA | NA | 450 | 37.5 | 58 (12.9); 392 (87.1) | 39 | 20 | NA | NA | NA | NA |
| 64 | Zhang et al B | 2020 | China | 12 | PHQ-4 | PHQ-4 | NA | NA | NA | NA | 927 | NA | 249 (26.9); 678 (73.1) | 113 | 121 | NA | NA | NA | NA |
| 65 | Zhang et al C | 2020 | China | 14 | PHQ‐9 | GAD‐7 | NA | NA | NA | NA | 524 | NA | 134 (25.6); 390 (74.4) | 164 | 206 | NA | NA | NA | NA |
| 66 | Zhang et al D | 2020 | China | 14 | NA | NA | NA | NA | NA | NA | 107 | 30.3(5.5) | 10 (9.3);  97 (90.7) | NA | NA | NA | NA | NA | NA |
| 67 | Zhang et al E | 2020 | China | 12 | PHQ‐9 | GAD‐7 | NA | NA | NA | NA | 1563 | NA | 270 (17.3); 1293 (82.7) | 792 | 699 | NA | NA | NA | NA |
| 68 | Zhang et al F | 2020 | China | 16 | HADS | HADS | NA | NA | NA | NA | 642 | NA | 96 (15.0); 546 (85.0) | 472 | 275 | NA | NA | NA | NA |
| 69 | Zhang et al G | 2021 | China | 14 | NA | NA | NA | NA | NA | NA | 946 | 33 (28,39) | 276 (29.2); 670 (70.8) | NA | NA | NA | NA | NA | NA |
| 70 | Zheng et al | 2021 | China | 16 | SDS | SAS | NA | NA | NA | NA | 3228 | NA | 107 (3.3); 3121 (96.7) | 1107 | 585 | NA | NA | NA | NA |
| 71 | Zhou et al A | 2020 | China | 13 | PHQ‐9 | GAD‐7 | NA | NA | NA | NA | 606 | 33.8 (8.1) | 114 (18.8); 492 (81.2) | 349 | 275 | NA | NA | NA | NA |
| 72 | Zhou et al B | 2021 | China | 12 | NA | NA | NA | NA | ProQOL Scale | NA | 1734 | 33.3 (6.4) | 429 (24.7); 1305 (75.3) | NA | NA | NA | NA | 527 | NA |
| 73 | Zhu et al A | 2020 | China | 13 | SDS | SAS | NA | NA | NA | NA | 165 | 34.2 (8.1) | 28 (17.0); 137 (83.0) | 73 | 33 | NA | NA | NA | NA |
| 74 | Zhu et al B | 2020 | China | 15 | NA | SAS | NA | NA | NA | NA | 453 | NA | 23 (5.1);  430 (94.9) | NA | 185 | NA | NA | NA | NA |
| 75 | Arafa et al | 2021 | Egypt and Saudi Arabia | 17 | DASS-21 | DASS-21 | DASS-21 | NA | NA | NA | 426 | NA | 214(50.2); 212 (49.8) | 294 | 251 | 238 | NA | NA | NA |
| 76 | Chatterjee et al | 2020 | India | 12 | DASS-21 | DASS-21 | DASS-21 | NA | NA | NA | 152 | 42.1 (12.2) | 119 (78.3); 33 (21.7) | 53 | 60 | 50 | NA | NA | NA |
| 77 | Grover et al | 2020 | India | 12 | DASS-21 | DASS-21 | DASS-21 | NA | NA | NA | 144 | 41.1 (8.6) | 66 (45.8); 78 (54.2) | 76 | 74 | 20 | NA | NA | NA |
| 78 | Gupta et al A | 2020 | India | 14 | NA | GAD‐7 | NA | NA | NA | NA | 368 | NA | 168 (45.7); 200 (54.3) | NA | 181 | NA | NA | NA | NA |
| 79 | Gupta et al B | 2020 | India | 17 | HADS | HADS | NA | NA | NA | NA | 749 | NA | 556 (74.2); 193 (25.8) | 211 | 264 | NA | NA | NA | NA |
| 80 | Gupta et al C | 2020 | India | 17 | HADS | HADS | NA | NA | NA | NA | 1124 | NA | 718 (63.9); 406 (36.1) | 354 | 418 | NA | NA | NA | NA |
| 81 | Jain et al | 2020 | India | 14 | NA | GAD‐7 | NA | NA | NA | NA | 512 | NA | 285 (55.7); 227 (44.3) | NA | 380 | NA | NA | NA | NA |
| 82 | Jose et al | 2020 | India | 14 | NA | NA | NA | NA | NA | CD-RISC | 120 | 29.0 (4.4) | 32 (26.7); 88(73.3) | NA | NA | NA | NA | NA | 19 |
| 83 | Khanna et al | 2020 | India | 12 | PHQ‐9 | NA | NA | NA | NA | NA | 2355 | 42.5 (12.1); 40.0 (25-82) | 1332 (56.7); 1018 (43.2) | 765 | NA | NA | NA | NA | NA |
| 84 | Khasne et al | 2020 | India | 10 | NA | NA | NA | NA | NA | NA | 2026 | NA | 1117 (55.0); 909 (45.0) | NA | NA | NA | NA | NA | NA |
| 85 | Mathur et al | 2020 | India | 12 | DASS-21 | DASS-21 | DASS-21 | NA | NA | NA | 200 | 42.1 (12.2) | 138 (69.0); 62 (31.0) | 34 | 39 | 19 | NA | NA | NA |
| 86 | Patel et al | 2021 | India | 10 | DASS-21 | DASS-21 | PSS | NA | NA | NA | 302 | NA | 189 (62.6); 113 (37.4) | 56 | 60 | 201 | NA | NA | NA |
| 87 | Podder et al | 2020 | India | 12 | NA | NA | PSS-10 | NA | NA | NA | 384 | 33.7 (9.3); 30.8 (7.8) | 213 (55.5); 171 (44.5) | NA | NA | 330 | NA | NA | NA |
| 88 | Sunil et al | 2021 | India | 14 | NA | NA | PSS-10 | NA | NA | NA | 313 | NA | 111 (35.5); 202 (64.5) | NA | NA | 169 | NA | NA | NA |
| 89 | Suryavanshi et al | 2020 | India | 14 | PHQ‐9 | GAD‐7 | NA | NA | NA | NA | 197 | NA | 96 (48.7); 101 (51.2) | 82 | 99 | NA | NA | NA | NA |
| 90 | Wilson et al | 2020 | India | 14 | PHQ‐9 | GAD‐7 | PSS-10 | NA | NA | NA | 350 | 30.2 (5.2) | 187 (53.4); 163 (46.6) | 173 | 232 | 289 | NA | NA | NA |
| 91 | Margaretha et al | 2020 | Indonesia | 11 | DASS-21 | DASS-21 | DASS-21 | NA | NA | NA | 682 | NA | 192 (28.2); 490 (71.8) | 117 | 228 | 216 | NA | NA | NA |
| 92 | Nasrullah et al | 2021 | Indonesia | 14 | DASS-21 | DASS-21 | DASS-21 | NA | NA | NA | 644 | NA | 160 (24.8); 484 (75.2) | 151 | 423 | 354 | NA | NA | NA |
| 93 | Setiawati et al | 2021 | Indonesia | 14 | NA | STAI | NA | NA | NA | NA | 227 | 39.7 (9.4); 38 (23-58) | 38 (16.7); 189 (83.3) | NA | 137 | NA | NA | NA | NA |
| 94 | Sujadi et al | 2020 | Indonesia | 13 | NA | GAD‐7 | NA | NA | NA | NA | 501 | NA | 213 (42.5); 288 (57.5) | NA | 334 | NA | NA | NA | NA |
| 95 | Hassannia et al | 2020 | Iran | 14 | HADS | HADS | NA | NA | NA | NA | 487 | NA | NA | 235 | 306 | NA | NA | NA | NA |
| 96 | Moayed et al | 2021 | Iran | 14 | NA | NA | DASS-21 | NA | NA | NA | 217 | 39.6 | 111 (51.2); 106 (48.8) | NA | NA | 217 | NA | NA | NA |
| 97 | Pouralizadeh et al | 2020 | Iran | 15 | PHQ‐9 | GAD‐7 | NA | NA | NA | NA | 441 | 36.3 (8.7) | 21 (4.8);  420 (95.2) | 313 | 324 | NA | NA | NA | NA |
| 98 | Karim et al | 2020 | Iraq | 13 | NA | HAQ | NA | NA | NA | NA | 402 | NA | NA | NA | 164 | NA | NA | NA | NA |
| 99 | Awano et al | 2020 | Japan | 14 | CES-D | NA | NA | NA | NA | NA | 848 | 37 (28-47) | 213 (25.1); 635 (74.9) | 237 | NA | NA | NA | NA | NA |
| 100 | Ide et al | 2021 | Japan | 14 | NA | NA | NA | NA | NA | NA | 2697 | NA | 702 (26.0); 1995 (74.0) | NA | NA | NA | NA | NA | NA |
| 101 | Yamamoto et al | 2020 | Japan | 12 | NA | NA | NA | NA | NA | NA | 661 | NA | NA | NA | NA | NA | NA | NA | NA |
| 102 | Naser et al | 2020 | Jordan | 15 | PHQ‐9 | GAD‐7 | NA | NA | NA | NA | 1163 | NA | 510 (43.9); 653 (56.1) | 907 | 823 | NA | NA | NA | NA |
| 103 | Shahrour et al | 2020 | Jordan | 17 | NA | NA | SASRQ | NA | NA | NA | 448 | 32.0 (8.0) | 120 (26.8); 328 (73.2) | NA | NA | 287 | NA | NA | NA |
| 104 | Park et al | 2020 | Korea | 14 | DASS-21 | DASS-21 | DASS-21 | NA | MBI-HSS | NA | 115 | 41 (37-38) | 48 (41.7);  67 (58.3) | 20 | 23 | 5 | NA | 104 | NA |
| 105 | Fauzi et al | 2020 | Malaysia | 14 | DASS-21 | DASS-21 | DASS-21 | NA | NA | NA | 1050 | 33.1 (7.0) | 299 (28.5); 751 (71.5) | 325 | 312 | 247 | NA | NA | NA |
| 106 | Woon et al | 2020 | Malaysia | 14 | DASS-21 | DASS-21 | DASS-21 | NA | NA | NA | 399 | NA | 107 (26.8); 292 (73.2) | 88 | 126 | 116 | NA | NA | NA |
| 107 | Zakaria et al | 2021 | Malaysia | 11 | NA | NA | NA | NA | Adopted Queationnaire | NA | 216 | 30 | 68 (31.5); 148 (68.5) | NA | NA | NA | NA | 184 | NA |
| 108 | Kafle et al | 2021 | Nepal | 16 | NA | NA | NA | NA | NA | NA | 254 | 26.0 (4.5) | 128 (50.4); 126 (49.6) | NA | NA | NA | NA | NA | NA |
| 109 | Khanal et al | 2020 | Nepal | 16 | HADS | HADS | NA | NA | NA | NA | 475 | 28.2 (5.8) | 225 (47.4); 250 (52.6) | 178 | 199 | NA | NA | NA | NA |
| 110 | Pandey et al | 2021 | Nepal | 15 | DASS-21 | DASS-21 | DASS-21 | NA | NA | NA | 404 | 32.3 (8.2) | 147 (36.4); 257 (63.6) | 117 | 144 | 69 | NA | NA | NA |
| 111 | Shrestha et al | 2020 | Nepal | 10 | NA | GAD‐7 | NA | NA | NA | NA | 101 | NA | 43 (42.6);  58 (57.4) | NA | 74 | NA | NA | NA | NA |
| 112 | Alshekaili et al | 2020 | Oman | 18 | DASS | DASS-21 | DASS-21 | NA | NA | NA | 1139 | 36.3 (6.5) | 228 (20.0); 911 (80.0) | 368 | 388 | 271 | NA | NA | NA |
| 113 | Khamis et al | 2020 | Oman | 16 | WHO-5 | GAD‐7 | PSS-10 | NA | NA | NA | 402 | 36.4 (6.7) | 0 (0.0);  402 (100) | 182 | 271 | 215 | NA | NA | NA |
| 114 | Arshad et al AR | 2020 | Pakistan | 13 | NA | GAD‐7 | NA | NA | NA | NA | 431 | NA | 238 (55.2); 193 (44.8) | NA | 265 | NA | NA | NA | NA |
| 115 | Arshad et al B | 2020 | Pakistan | 14 | DASS-21 | DASS-21 | DASS-21 | NA | NA | NA | 276 | NA | 182 (65.9); 94 (34.1) | 28 | 70 | 20 | NA | NA | NA |
| 116 | Hasan et al S | 2020 | Pakistan | 12 | NA | GAD‐7 | NA | NA | NA | NA | 151 | 29.0 (7.3) | 66 (43.7);  85 (56.3) | NA | 96 | NA | NA | NA | NA |
| 117 | Imran et al | 2020 | Pakistan | 14 | PHQ‐9 | GAD‐7 | SASRQ | NA | NA | NA | 10178 | 31.5 (6.9) | 4402 (43.3); 5776 (56.7) | 4696 | 4132 | 448 | NA | NA | NA |
| 118 | Kumar et al | 2021 | Pakistan | 11 | DASS-21 | DASS-21 | DASS-21 | NA | NA | NA | 224 | NA | 170 (75.9); 54 (24.1) | 72 | 62 | 59 | NA | NA | NA |
| 119 | Sandesh et al | 2020 | Pakistan | 10 | DASS-21 | DASS-21 | DASS-21 | NA | NA | NA | 112 | NA | 64 (57.1);  48 (42.9) | 101 | 107 | 108 | NA | NA | NA |
| 120 | Labrague et al A | 2020 | Phillipines | 15 | NA | CAS | NA | NA | NA | NA | 325 | 30.9 (6.7) | 82 (25.2); 243 (74.8) | NA | 123 | NA | NA | NA | NA |
| 121 | Labrague et al B | 2020 | Phillipines | 16 | NA | CAS | NA | NA | NA | NA | 736 | 31.9 (7.4) | 162 (22.0); 574 (78.0) | NA | 402 | NA | NA | NA | NA |
| 122 | Khoodoruth et al | 2021 | Qatar | 14 | DASS-21 | DASS-21 | DASS-21 | NA | NA | NA | 127 | NA | 79 (62.2);  48 (37.8) | 54 | 53 | 39 | NA | NA | NA |
| 123 | AbuSnieneh et al | 2021 | Saudi Arabia | 14 | PHQ‐9 | GAD‐7 | NA | NA | NA | NA | 1265 | 28.8 (5.3) | 164 (13.0); 1101(87.0) | 754 | 629 | NA | NA | NA | NA |
| 124 | Al Ammari et al | 2021 | Saudi Arabia | 17 | PHQ‐9 | GAD‐7 | NA | NA | NA | NA | 720 | NA | 258(35.8); 462(64.2) | 354 | 357 | NA | NA | NA | NA |
| 125 | Alenazi et al | 2020 | Saudi Arabia | 11 | NA | Dispositional cancer worry scale | NA | NA | NA | NA | 4920 | 34.1 (8.1) | 2307 (46.9); 2613 (53.1) | NA | 3368 | NA | NA | NA | NA |
| 126 | Al-Hanawi et al | 2020 | Saudi Arabia | 14 | NA | NA | NA | NA | NA | NA | 950 | NA | NA | NA | NA | NA | NA | NA | NA |
| 127 | Almater et al | 2020 | Saudi Arabia | 14 | PHQ‐9 | GAD‐7 | PSS | NA | NA | NA | 107 | 32.9 (9.6) | 60 (56.1); 47(43.9) | 56 | 50 | 77 | NA | NA | NA |
| 128 | Alsulimani et al | 2021 | Saudi Arabia | 14 | NA | NA | NA | NA | NA | NA | 646 | 34.1 (9.5) | 248 (38.4); 398 (61.6) | NA | NA | NA | NA | NA | NA |
| 129 | Alzaid et al | 2020 | Saudi Arabia | 14 | NA | GAD‐7 | NA | NA | NA | NA | 441 | NA | 120 (27.2); 321 (72.8) | NA | 212 | NA | NA | NA | NA |
| 130 | Balay-odao et al | 2021 | Saudi Arabia | 15 | DASS-21 | DASS-21 | DASS-21 | NA | NA | NA | 281 | 33.3 (6.4) | 40 (14.2); 241 (85.8) | 137 | 179 | 50 | NA | NA | NA |
| 131 | Khanagar et al | 2020 | Saudi Arabia | 14 | DASS-21 | DASS-21 | DASS-21 | NA | NA | NA | 110 | 25.1 | 39 (35.5);  71 (64.5 ) | 12 | 8 | 1 | NA | NA | NA |
| 132 | Temsah et al | 2020 | Saudi Arabia | 13 | NA | GAD‐7 | NA | NA | NA | NA | 582 | 36.0 (8.5) | 145 (24.9); 437 (75.1) | NA | 185 | NA | NA | NA | NA |
| 133 | Tan et al | 2020 | Singapore | 16 | HADS | HADS | SAQ | NA | OLBI | NA | 3075 | 36.8 (10.0) | 794 (25.8); 2199 (71.5) | 979 | 1253 | 205 | NA | 2097 | NA |
| 134 | Teo et al | 2020 | Singapore | 12 | SDS | GAD‐7 | NA | NRS | NA | NA | 122 | 34 (21-73) | 32 (26.2); 90 (73.8) | 64 | 55 | NA | 89 | NA | NA |
| 135 | Chew et al B | 2020 | Singapore and India | 12 | DASS-21 | DASS-21 | DASS-21 | NA | NA | NA | 906 | 29 (25-35) | 323 (35.7); 583 (64.3) | 96 | 142 | 47 | NA | NA | NA |
| 136 | Chen et al R | 2021 | Taiwan | 15 | NA | NA | NA | NA | NA | NA | 12596 | 33.1 (7.5) | 555 (4.4); 12041 (95.6) | NA | NA | NA | NA | NA | NA |
| 137 | Aksoy et al | 2020 | Turkey | 15 | NA | STAI; IUS-12 | NA | NA | NA | NA | 758 | 30.5( 7.2) | 55(7.3); 703(92.7) | NA | 275 | NA | NA | NA | NA |
| 138 | Alan et al | 2020 | Turkey | 14 | DASS-21 | DASS-21 | DASS | NA | NA | NA | 416 | 33.6 (8.7) | 87 (20.9); 329 (79.1) | 274 | 257 | 238 | NA | NA | NA |
| 139 | Bahadir-Yilmaz et al | 2020 | Turkey | 14 | STAI | STAI | STAI | STAI | NA | NA | 1457 | NA | 277 (19.0); 1180 (81.0) | 338 | 955 | 1072 | 1111 | NA | NA |
| 140 | Çalişkan et al | 2020 | Turkey | 14 | HADS | HADS | NA | NA | NA | NA | 290 | 31.8 (6.9) | 179 (61.7); 111(38.3) | 180 | 103 | NA | NA | NA | NA |
| 141 | Erdem et al | 2021 | Turkey | 10 | NA | BAS | NA | NA | NA | NA | 121 | NA | 75 (62.0); 46 (38.0) | NA | 44 | NA | NA | NA | NA |
| 142 | Karasu et al | 2021 | Turkey | 12 | NA | TAI | NA | NA | NA | NA | 710 | 33.9 (8.6) | 279 (39.3); 431 (60.7) | NA | 704 | NA | NA | NA | NA |
| 143 | Sahin et al | 2020 | Turkey | 11 | PHQ‐9 | GAD‐7 | NA | NA | NA | NA | 939 | NA | 319 (34.0); 620 (66.0) | 729 | 565 | NA | NA | NA | NA |
| 144 | Uyaroglu et al | 2020 | Turkey | 11 | NA | GAD‐7 | NA | NA | NA | NA | 113 | 29 (5) | 60 (53.1); 53 (46.9) | NA | 56 | NA | NA | NA | NA |
| 145 | Yıldırım et al | 2021 | Turkey | 10 | DASS-21 | DASS-21 | DASS-21 | NA | NA | NA | 245 | 33.2 (7.3) | 121 (49.4); 124 (50.6) | 50 | 44 | 24 | NA | NA | NA |
| 146 | Yilmaz et al | 2020 | Turkey | 12 | NA | BAS | NA | NA | NA | NA | 240 | NA | 220 (91.7); 20 (8.3) | NA | 62 | NA | NA | NA | NA |
| 147 | Yoruk et al | 2021 | Turkey | 12 | BDI | NA | NA | NA | NA | NA | 377 | 32.2 (8.1) | NA | 120 | NA | NA | NA | NA | NA |
| 148 | Than et al | 2020 | Vietnam | 12 | DASS-21 | DASS-21 | DASS-21 | NA | NA | NA | 173 | 31 (27-36) | 55 (31.8); 118 (68.2) | 35 | 58 | 22 | NA | NA | NA |
| *Asian Pacific involved China, Saudi Arabia, Taiwan | | | | | | | | | | | | | | | | | |  |  |
| **Asian Pacific involved India, Indonesia, Singapore, Malaysia and Vietnam | | | | | | | | | | | | | | | | | |  |  |
| ***Questionnaire form was adopted from Michelle Post, Public Welfare, Vol. 39, No. 1, 1981, American Public Welfare Association | | | | | | | | | | | | | | | | | |  |  |
|  | | | | | | | | | | | | | | | | | |  |  |
| Abbreviation: | | | | | | | | | | | | | | | | | |  |  |
| Asi-3: Anxiety Sensitivity Index-3; | | | | | | | | | | | | | | | | | |  |  |
| BAS: Beck Anxiety Scales; | | | | | | | | | | | | | | | | | |  |  |
| BDI: Beck Depression Inventory; | | | | | | | | | | | | | | | | | |  |  |
| CAS: Coronavirus Anxiety Scale; | | | | | | | | | | | | | | | | | |  |  |
| CCMD-3: Chinese Classification and the Diagnose Criterion of Mental Disorder; | | | | | | | | | | | | | | | | | |  |  |
| CD-RISC: Connor-Davidson Resilience Scale; | | | | | | | | | | | | | | | | | |  |  |
| CES-D: Center for Epidemiology Studies-Depression; | | | | | | | | | | | | | | | | | |  |  |
| DASS-21: Depression-Anxiety-Stress Scale-21; | | | | | | | | | | | | | | | | | |  |  |
| FCV-19S: Fear of Coronavirus 2019 Scale; | | | | | | | | | | | | | | | | | |  |  |
| FS-HPs: Fear Scale for Healthcare Professionals; | | | | | | | | | | | | | | | | | |  |  |
| GAD-2: 2-item Generalized Anxiety Disorder; | | | | | | | | | | | | | | | | | |  |  |
| GAD-7: 7-item Generalized Anxiety Disorder; | | | | | | | | | | | | | | | | | |  |  |
| HADS: Hospital Anxiety and Depression Scale 14 items; | | | | | | | | | | | | | | | | | |  |  |
| HAMA: Hamilton Anxiety Rating Scale; | | | | | | | | | | | | | | | | | |  |  |
| HAMD: Hamilton Depression Rating Scale; | | | | | | | | | | | | | | | | | |  |  |
| IUS-12: Intolerance of Uncertainty Scale; | | | | | | | | | | | | | | | | | |  |  |
| MBI: Maslach Burnout Inventory; | | | | | | | | | | | | | | | | | |  |  |
| MBI-HSS: Maslach Burnout Inventory- Human Service Survey | | | | | | | | | | | | | | | | | |  |  |
| MINI: Mini International Neuropsychiatric Interview; | | | | | | | | | | | | | | | | | |  |  |
| NRS: Numerical rating scale | | | | | | | | | | | | | | | | | |  |  |
| OLBI: Oldenburg Burnout Inventory; | | | | | | | | | | | | | | | | | |  |  |
| PHQ-2: 2-item Patient Health Questionnaire; | | | | | | | | | | | | | | | | | |  |  |
| PHQ-4: Patient Health Questionnaire-4; | | | | | | | | | | | | | | | | | |  |  |
| PHQ-9: 9-item Patient Health Questionnaire; | | | | | | | | | | | | | | | | | |  |  |
| PSS: Perceived Stress Scale; | | | | | | | | | | | | | | | | | |  |  |
| SAQ: Safety Attitudes Questionnaire; | | | | | | | | | | | | | | | | | |  |  |
| SAS: Zung's Self-Rating Anxiety Scale; | | | | | | | | | | | | | | | | | |  |  |
| SASRQ: Stanford Acute Stress Reaction Questionnaire; | | | | | | | | | | | | | | | | | |  |  |
| SDS: Zung's Self-rating Depression Scale; | | | | | | | | | | | | | | | | | |  |  |
| STAI: State and Trait Anxiety Inventory; | | | | | | | | | | | | | | | | | |  |  |
| WHO-5: WHO Well-being Index; | | | | | | | | | | | | | | | | | |  |  |
| NA: Not available; | | | | | | | | | | | | | | | | | |  |  |
