# Supplementary material for: Psychological distress among healthcare providers during COVID-19 in Asia: Systematic review and meta-analysis
Source: PLoS One. 2021 Oct 14;16(10):e0257983. doi: 10.1371/journal.pone.0257983 (PMC8516240; doi:10.1371/journal.pone.0257983)
Supplement: Attachment — Submitted filename: Comments.docx [file pone.0257983.s012.docx]

The topic is interesting and contains useful information for readers, although the following comments will help reinforce this manuscript.

1- - In the abstract, name, the used databases.
2- - Please in introduction, highlighted which factors affect the psychological distress of HCW based on quantitative and especially qualitative studies conducted in vulnerable countries in Asia?
3- - exactly mention who do you mean by HCW?
4- Please do a search on the Web of Sciences database.
5- Please specify the inclusion and exclusion criteria clearly.
6- Did you do a manual search (based on Google Scholar citation or check the references of each article)
7- Please improve the quality of Forest plots (All Figures).
8- Please discuss the causes of high heterogeneity in this study (in discussion section).
9- Please explain the reason for publication bias in this study (in discussion section).
